# Postoperative Care of the Maxillofacial Surgery Patient

**DOI:** 10.1007/978-981-15-1346-6_12

**Published:** 2020-06-24

**Authors:** J. Naveen Kumar, Poornima Ravi

**Affiliations:** 1 Bhagwan Mahaveer Jain hospital, Bangalore, India; 2 Associate Professor, SRM Dental College, Ramapuram, Chennai, Tamil Nadu India; 3 Ananthapuri Hospitals & Research Institute, Kerala Institute of Medical Sciences, Trivandrum, Kerala India; 4 Department of Maxillofacial Plastic Surgery, Uppsala University Hospital, Uppsala, Sweden; 5 Associate Professor, Department of Dentistry, All India Institute of Medical Sciences, Bhopal, Madhya Pradesh India; 6 Oral and Maxillofacial Surgery, Sri Ramachandra Institute of Higher Education and Research, Chennai, India; 7 Department of Oral and Maxillofacial Surgery, SRM Dental College, Ramapuram, Chennai, Tamil Nadu India

**Keywords:** Postoperative care, Perioperative care, Maxillofacial ward, Extubation, Nutrition, Mobilization, Airway, Fever, Nausea, Vomiting, General anesthesia

## Abstract

“Surgery” is defined as “*treatment of injuries or disorders of the body by incision or manipulation, especially with instruments*”. As such, it is nothing more than the mere performance of maneuvers at the operating room and certainly does not qualify to be called “treatment”. It is postoperative care that completes the process, ultimately benefitting the patient. In general, this includes the overall maintenance of wellbeing and early recovery of function before the patient can be discharged to be on his own. Additionally, the maxillofacial patient presents with issues exclusive to the anatomy and physiology of the head and neck region. One needs to have in-depth knowledge of these unique aspects, in addition to being a shrewd clinician at the postoperative ward. This chapter aims to equip the surgeon with such information as is necessary to provide the best of services following maxillofacial surgery.

Postoperative care of the patient encompasses the time from the completion of the surgical procedure to the complete return of the patient to the normal physiological state. This is divided into three phases. Phase I is early recovery and takes place in the postanesthesia care unit (PACU). Phase II is intermediate recovery, and takes place in the ward. Phase III is late recovery that occurs after discharge.

## Assessment of the Patient After Surgery

### Assessment of the Patient Immediately After Surgery

Postoperative care of the patient begins immediately after the surgical procedure has been completed, even before the anesthesia is reversed. The first step is clearing the airway of blood and debris. Maxillomandibular fixation and occlusal splints, if placed earlier need to removed [1]. The next step is the removal of the throat pack.

#### Care of the Airway

The decision to extubate or not must be made in conjunction with the anesthetist. Cases in which there is a high risk of airway edema will require the ET tube to be retained [1]. These include:

- Space infections compressing on the airway, e.g. Ludwig’s angina.
- Severe facial trauma where there is likelihood of blood ooze, edema, or tongue fall back.
- Prolonged surgery, e.g. Free flap reconstructions.

In other cases, the patient may be extubated on the table. Awake extubation is usually preferred for head and neck surgery. The patient may be extubated when the following criteria are met [1]:

- No bleed from surgical site or secretion in the oropharynx.
- Patient is able to follow verbal commands.
- Patient is able to sustain head lift for at least 5 seconds.
- Patient is breathing on his own, with respiratory rate less than 24/min, tidal volume greater than 5 ml/kg, and Spo2 > 90%.

Once the extubation is done, an oropharyngeal airway can be inserted to prevent clenching of teeth and the tongue from falling back, which can cause obstruction. Alternatively, a nasopharyngeal airway can be used in case the surgeon deems oral cavity unfit for manipulation using the former. This must remain in place until the patient is conscious and obeys commands.

Some patients, such as those who have undergone extensive face and neck resection, may have required a tracheostomy prior to surgery. The tube must be secured after surgery by taping the stay sutures to the neck or chest. Tracheostomy care in the postoperative period is critical; a blocked or dislodged tube can have disastrous consequences [2].

1. The tube must be checked at frequent intervals.
2. The tube stoma must be covered with a humidifying bib, or a moist gauze.
3. The tube must be suctioned regularly to prevent clogging due to secretions. The suction catheter must be inserted to half of its length, which will correspond to the carina (Patients usually cough when this point is reached). Suction is then applied while simultaneously withdrawing the catheter.
4. To break up secretions, small quantities of sterile water can be syringed in and suctioned immediately.

#### Need for Ventilation in the PACU

In some instances, ventilation may be required even after anesthetic recovery. Some examples include:

1. Patients who have history of COPD.
2. Severe trauma or infection.

While monitoring the patient who is on a ventilator, it is important to be aware of the various modes that the ventilator operates on [3]. These have been summarized in Table 12.1. Patients who have been on the ventilator for long periods of time need to be weaned off slowly. OMF surgeons frequently come across such patients in neurosurgical ward having concomitant traumatic brain injuries and craniomaxillofacial fractures. This may be done by setting the ventilator in CPAP mode (Continuous Positive Airway Pressure). This allows the patient to breathe, with the ventilator taking over if the patient is unable to do so.

**Table 12.1:** Ventilator modes and settings

Ventilator mode	Type of setting	Description/indications
Volume control	Continuous mandatory ventilation (CMV) Synchronous intermittent mandatory ventilation (SIMV)	Patients with respiratory muscle weakness or LV dysfunction If patient breathes rapidly, may cause hyperinflation and respiratory alkalosis Patient breathes partially on their own; meant for patients who breathe rapidly on CMV Mandatory breaths are synchronized with spontaneous breathing
Pressure control	Pressure controlled ventilation (PCV) Pressure support ventilation	Patients with neuromuscular disease but normal lungs who can control volume. Used during weaning, patient determines respiratory volume and frequency, but the ventilator provided continuous positive airway pressure (CPAP)

#### Monitoring in the PACU

Once the patient has been shifted to the recovery room, the cardiac monitor and pulse oximeter must be attached for proper monitoring. The following parameters must be monitored continuously:

- *Oxygen Saturation:* Hypoxia can occur in the postoperative period and the patient must be kept on oxygen for 1–2 h (2–6 L/min) to prevent this. Oxygen may be delivered using a face mask or through nasal prongs.
- *Pulse, blood pressure:* Increase in these parameters may indicate pain. Serious complications (Infarction, Malignant hyperthermia) may produce a drastic change in these parameters and must be recognized.
- *ECG waveform:* To monitor cardiac status.
- *Postanesthesia tremors/shivering:* This can occur on the table during recovery from anesthesia. It can occur if the patient is hypothermic, and is commonly associated with the use of halogenated anesthetics. Management consists of rewarming the patient. Tramadol and meperidine may be used to stop uncontrollable shivering [4].

The suction apparatus must be kept handy to evacuate blood ooze or secretions that may hamper the airway. It is advisable to avoid Maxillomandibular fixation (MMF) in the immediate postoperative period; if required, this may be done after 24 h.

Detailed surgical notes must also be recorded, along with the number and type of implants that were used. Postoperative instructions must also be documented in detail. A list of notes to be completed by the surgeon before leaving the operation theater complex is summarized in Table 12.2.

**Table 12.2:** Checklist for completion before leaving the theater complex

Checklist to complete before leaving the OT	
Surgical notes Details of implants and hardware used Postoperative instructions to be followed by nursing staff Postoperative fluid management instructions Postoperative medication dose and schedule Biopsy requisition form Requisition form for aspirates/swabs and others Investigation requisition form	

#### Briefing the Patient and Family

Immediately after the surgery, the surgeon must interact with the patient’s immediate caregivers, giving them the details of the procedure, and any anticipated complications.

#### Discharge from PACU to Ward

This is done when the patient has regained consciousness, with adequate respiratory function and stable vitals. Decisions can be made based on a standard scoring system, such as the Aldrete scoring system [5]. The scoring system which was originally proposed in 1970, underwent modifications in 1995 and 1999. The various factors considered are patient activity, respiration, circulation, consciousness, O_2_ saturation, pain, surgical site bleeding, and nausea/vomiting. Patients scoring greater than 9 on this scale can be moved to the ward for the next phase of care. Readers are advised to refer the article for getting a detailed idea of the scoring table.

### Comprehensive Assessment of the Patient in the Ward

This is done according to the SOAP format [6]. SOAP is an acronym for Subjective, Objective, Assessment, and Plan. In Subjective evaluation, the patient must be asked if they have any complaints. Specific complaints are recorded. In Objective evaluation, a thorough evaluation of the patient is done by the physician. This includes evaluation of the vital signs, fluid intake and output, as well as an assessment of the surgical site. Helpful information may be obtained from the TPR chart, input/output chart, and nurses’ notes. Based on the subjective and objective evaluation, the patient’s current status is assessed (Tables 12.3 and 12.4). This is used to formulate a plan.

**Table 12.3:** Subjective assessment of the patient

Subjective parameters evaluated	Assessment made
Pain (use visual analog scale- VAS/faces scale)	Whether the pain medication is adequate or whether it needs to be increased or discontinued (see Sect. 12.2.2.1)
Nausea/vomiting	If the patient has experienced this, assess the need for antiemetics, and insertion of nasogastric tube to decompress the stomach. (Sect. 12.2.5.5)
Mobility	The patient must be encouraged to sit up and walk by the first postoperative day. (Section IID)
Function- e.g. swallowing, speech, nerve function	It is important to evaluate branches of the facial nerve and trigeminal nerve that may have been at risk of damage during surgery
Passing urine/stools/flatulence	Inability to pass urine may indicate inadequate fluid therapy, and may lead to acute renal failure. (see Sect. 12.2.5.6) Inability to pass stools may be a sign of paralytic ileus; may occur after iliac crest harvest

**Table 12.4:** Objective assessment of patient

Objective parameters evaluated	Assessment made
*Vital signs*	
Temperature Pulse Blood pressure Respiratory rate	Is postoperative fever present? If so, it must be worked up (Sect. 12.2.5.2) Changes in pulse and blood pressure have several causes (Sect. 12.2.5.3) If abnormal, evaluate whether the patient has respiratory distress (Sect. 12.2.5.4)
*Fluid input/output*	
	Is the input adequate? (Sect. 12.2.1) Is urinary output adequate? This helps assess renal function(Sect. 12.2.5.6)
*Surgical site evaluation*	
	The wound must be inspected, and assessed for healing. (Sect. 12.3)

#### Postoperative Investigations

Sometimes investigations may be required in the postoperative period, either to check the health status of the patient or to confirm the diagnosis of certain complications. A list of investigations that may be ordered and the indications for the same are summarized in Table 12.5.

**Table 12.5:** Postoperative investigations

Investigation	Indications
Hemoglobin Serum electrolytes WBC TC/DC Chest x-ray OPG/other facial radiographs	If there has been extensive blood loss during surgery, to determine the need for blood transfusion. Suspicion of electrolyte imbalances (seizures, palpitations, muscle cramps) Suspicion of spreading infection Suspicion of atelectasis/aspiration pneumonia To check the accuracy of reduction and status of plating.

## Formulating a Plan of Care Based on Assessment

### Fluid Therapy in the Postoperative Period

The patient is usually ‘nil per mouth’ for a few hours prior to surgery and after surgery. Apart from this, there is a loss of blood and body fluids in any surgery, which requires replacement. It is therefore essential to infuse intravenous fluids during this period [7, 8]. This is done for two purposes.

#### Replacement

Any fluid deficit that has occurred must be replaced by infusion. This could have occurred during either of the following periods:

- Preoperative period: This could be due to
  - NPO status.
  - Blood or fluid losses that may have occurred due to trauma, burns, etc.
- Intraoperative period: Surgical blood loss of greater than 500 ml or 7 ml/kg requires replacement.

#### Maintenance

This is to maintain the ongoing fluid requirements, till the patient resumes oral intake of fluids. Maintenance fluids are essential to maintain proper pH and electrolyte balance and for adequate organ perfusion.

#### Types of Fluids Used

There are three types of fluids than can be used—crystalloids, colloids, blood and blood products. The preference of one type of fluid over another has several controversies, and there are no clear-cut guidelines available [9]. A few indications for each fluid type are given below.

##### Crystalloids

Crystalloids are balanced salt solutions with or without the addition of a buffering agent. When infused into the bloodstream, crystalloids tend to leave the capillaries and enter the extravascular fluid compartment. Crystalloid infusion will increase fluid in the extravascular tissues and does little to expand the circulating blood volume. In maxillofacial surgery, crystalloids are favored as maintenance fluids during the postoperative period.

##### Colloids

Colloids are protein-containing solutions. Since these proteins have a large molecular size, under ordinary circumstances, these are prevented from crossing the capillary endothelial cells and going into the extravascular space. Therefore, they tend to expand vascular volume alone. Colloids are mostly used in the intraoperative period if there has been significant blood loss and the plasma volume needs to be expanded. It is not common to use colloids in the postoperative setting.

Commonly used iv fluids are listed in Table 12.6.

**Table 12.6:** Commonly used postoperative IV fluids

IV Fluid	Composition (in meq/l)	Indications	Risks
*Crystalloids*			
Lactated Ringers (RL)	Na −130; Cl −109; K −4; Ca −3; Lactate −28	Fluid of choice in postoperative maintenance	Lactic acidosis if liver function is poor
Dextrose Normal Saline (DNS)	Na −154; Cl −154; Dextrose −50 g	Alternative to RL	Hyperchloremic acidosis
Normal Saline (NS)	Na −154; Cl −154	Alternative to DNS in diabetics	Hyperchloremic acidosis
5% Dextrose (D5W)	Dextrose 50 g	Replacing free water deficit	Hyperglycemia in diabetics
*Colloids*			
Hetastarch	6% hydroxyethyl starch	Plasma volume expansion	Nephrotoxicity, coagulopathy

#### Strategy for Estimating Fluid Requirement

1. The input-output chart must be verified before determining the fluid requirement. This will help to estimate fluid excess (positive balance), or deficits (negative balance) and plan requirements.
2. Calculate the Estimated Fluid Requirement (EFR) for each hour:
  This is done using Holliday and Segar’s formula (The 4-2-1 rule) [10].
  First 10 kg: 4 cc/kg.
  Next 10 kg: 2 cc/kg.
  Above 20 kg: 1 cc/kg.
  E.g. a 60 kg adult will require (4 × 10) + (2 × 10) + (1 × 40) = 40 + 20 + 40 = 100 ml/h.
3. Calculate the total Estimated Fluid Deficit: This depends on the number of hours from the last oral intake to the next oral intake. For example, if the patient has not had oral intake for 12 h:
  EFD = EFR × no. of NPO hours = 100 × 12 = 1200 ml.
4. Estimate the surgical blood loss. If crystalloids are used to replace this blood loss, for a particular volume of blood, three times the volume of crystalloids are used for replacement. If colloids are used, the same volume is sufficient.
5. Estimate the amount of fluids that have already been infused during anesthesia.
6. Total postsurgical fluid requirement:
  EFD+(blood loss × 3)−fluids replaced during surgery.
  In the above scenario, if 300 ml of blood was lost, and one liter of fluid was infused during surgery, then:
  Total postsurgical fluid requirement:1200+ (300 × 3)−1000 = 1200 + 900−1000 = 1100 ml.

#### Liberal Versus Restrictive Fluid Therapy

In recent years, the above method of estimating fluid requirements has been criticized, as it tends to overestimate the amount of fluids needed by a patient [11]. Excessive fluid infusion may cause fluid shift into the extravascular compartment, which in turn can result in overload complications such as renal injury, acute respiratory distress syndrome, etc. On the other hand, liberal fluid infusion can reduce postoperative complications such as nausea, vomiting, and drowsiness.

For major systemic surgeries, the current trend is either to follow a ‘restrictive’ approach or a goal-directed therapy. Goal-directed therapy measures hemodynamic parameters such as stroke volume, and fluids are given accordingly. While this has been found useful in major surgeries, particularly abdominal surgeries, there is no evidence on its effectiveness in postoperative recovery for maxillofacial surgery. A liberal approach may be preferred for most kinds of maxillofacial surgery, which generally fall under low or intermediate risk procedures. Nevertheless, as long as postoperative fluids are being administered, the patient must be monitored for signs of overhydration such as peripheral edema, dyspnea, high blood pressure, and a bounding pulse. If any of these are present, the current fluid regimen must be reassessed [12].

#### Transfusion of Blood and Blood Products

Postoperative blood transfusion is rarely required in routine maxillofacial surgery. It has been stated that the risks of blood transfusion outweigh the benefits [13] (See Table 12.7), and currently a restrictive approach to blood transfusion is favored.

**Table 12.7:** Risks vs. benefits of blood transfusion

Risks of blood transfusion	Benefits of blood transfusion
Transmission of infections that cannot be identified by screening (cytomegalovirus, Epstein-Barr virus, B-19 parovirus, dengue, chikungunya, HHV-8, malarial parasite) Transfusion reactions	Better functional status Lower morbidity and mortality (for levels below 7.0 mg/dl)

If there has been extensive blood loss during surgery, or preexisting anemia, the postoperative hemoglobin must be assessed, and the decision to transfuse is based on this level [14]. This is described in Table 12.8.

**Table 12.8:** Indications for postoperative blood transfusion

Postoperative hemoglobin level	Risk factors/compensatory mechanisms present	Transfusion requirement
<6 g/dl		Required
6–8 g/dl	No risk factors	Not required
	Presence of risk factors (coronary artery disease, heart failure, cerebrovascular disease/limited mechanisms of compensation)	Required
	Presence of symptoms indicative of hypoxia (physiological transfusion triggers: Tachycardia, hypotension, electrocardiographic signs of ischemia, lactic acidosis, etc.)	Required
8–10 g/dl	Presence of symptoms indicative of hypoxia (physiological transfusion triggers: Tachycardia, hypotension, electrocardiographic signs of ischemia, lactic acidosis, etc.)	Required
	Absence of above symptoms	Not required
>10 g/dl		Not required

### Postoperative Medication

The maxillofacial surgeon must be aware of the type and dosage of medication that is required in the immediate postoperative period. Pain control and prophylaxis against infection are the most important factors to be kept in mind while prescribing medication.

#### Pain Control

Pain control is an important goal after every surgical procedure as it can not only affect the patients’ attitude, but it can also impair oxygenation and thereby delay wound healing. The pain must be assessed subjectively, by asking the patient to rate their pain on a standard scale (e.g. Visual Analogue scale or Faces pain scale). If the patient is in pain, pain medication must be increased or changed.

Preemptive analgesia is an evolving, controversial technique that involves the administration of analgesics prior to the onset of noxious stimuli. This is believed to limit the sensitization of the nervous system, thereby reducing the need for postoperative analgesia [15]. One effective preemptive technique is the infiltration of a long-acting local anesthetic, such as bupivacaine, into the incision site before closure. This provides effective pain relief throughout the postoperative period.

In the postoperative period, various classes of analgesics may be used [16]. Some of the commonly used analgesics are summarized in Table 12.9.

**Table 12.9:** Analgesics commonly used for postoperative pain control

Drug	Classification	Dosage and frequency	Precautions and adverse effects
Diclofenac	NSAIDS (aryl acetic acid derivatives)	75 mg bd adults 1.5 mg/kg bd children	Has been linked to adverse cardiovascular events—Avoid in patients with heart disease Gastric ulceration and bleeding may worsen Nephrotoxic; avoid in patients prone to kidney disease
Aceclofenac		100 mg bd adults Safety not established in children	Less than diclofenac, mild GI symptoms have been reported
Ketorolac	NSAIDS	20–30 mg q6hr 0.5 mg/kg q6hr	GI symptoms, hypertension reported in few patients
Acetaminophen (paracetamol)	NSAIDS (Para-amino phenol derivative)	0.5–1 g tds adults 10–15 mg/kg children	Hepatotoxicity
Tramadol	Synthetic opioid	50 mg bd or sos Safety not established in children.	May worsen nausea and vomiting. Like all opioids, respiratory depression is higher.
Morphine	Natural opioid	0.2–0.8 mg/kg bd	Respiratory depression, nausea, vomiting, constipation. Not suitable in immediate postop as consciousness cannot be evaluated

**Fig. 12.1:**
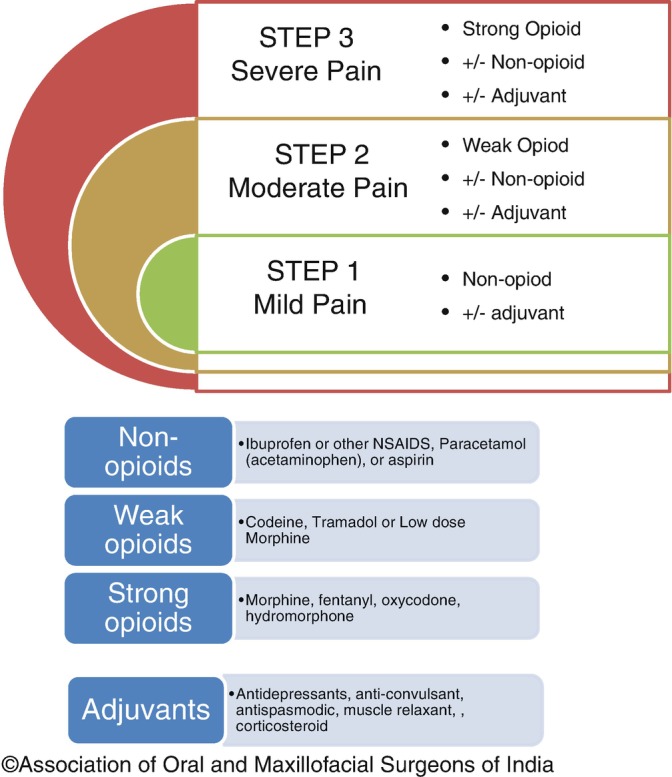
WHO analgesic ladder

The best method of choosing the appropriate analgesic is using the WHO analgesic ladder (Fig. 12.1). If the pain is not well controlled, the patient can move to the next step of the ladder. Once the pain is controlled, patients must be weaned by moving down the ladder [17]. This step ladder approach is just a broad lattice and has its own share of controversies and modifications. Readers are encouraged to read appropriate references for getting a broader picture of the analgesic ladder and a detailed discussion is beyond the scope of this chapter.

##### Patient-controlled Analgesia

Postoperative patients often require immediate pain relief at varied intervals. PCA allows the use of iv pumps which, when the patient presses a button, allows a bolus dose of analgesic to be delivered for immediate relief [18]. This allows analgesics to be tailored to the patients’ requirements and also records the amount of opioid being administered per day.

#### Anti-inflammatory Drugs

The role of corticosteroids in postoperative care is controversial. Corticosteroids are potent anti-inflammatory agents and are often used after surgery. It has been established that corticosteroids reduce pain and inflammation [19]. There is also evidence that single doses of steroids can reduce postoperative nausea and vomiting, and improve fatigue after surgery. However, the benefit of extended doses of steroids seems controversial and comes with the risk of impaired wound healing, infectious complications, and hyperglycemia. It is therefore recommended that steroids be used only till the first postoperative day. Dexamethasone 8 mg is generally used twice a day. For single day dosing, taper is not generally required.

Enzymatic anti-inflammatory medication is also used to reduce postoperative edema [20]. These include serratiopeptidase (10 mg tds) or trypsin:chymotrypsin (chymoral, 100,000 IU). However, its efficacy is based mostly on anecdotal reports and there is hardly any scientific evidence to back its use.

#### Antibiotic Prophylaxis

The use of antibiotic prophylaxis (for wound infections) in maxillofacial surgery is a controversial area. Literature evidence is insufficient to ascertain whether prophylaxis is required or not, and the duration of prophylaxis has no definite guidelines. Based on recent systematic reviews, Table 12.10 sums up current recommendations [21, 22].

**Table 12.10:** Recommendations for antibiotic prophylaxis

Type of surgery	Recommendation for antibiotic prophylaxis
Orthognathic surgery	Strongly recommended
Trauma—compound fractures	Recommended
Resections involving oral and cervical region	Recommended
Reconstruction	Weak evidence; physician judgment
Cleft surgery	Weak evidence; physician judgment
Minor oral surgery	Weak evidence; physician judgment

Preferred antibiotics for prophylaxis include:

- Amoxicillin-Clavulanate 1.2 mg IV bd.
- Amoxicillin 500 mg per oral tds (OR) Cefotaxime 1 g IV bd with Metronidazole 500 mg IV tds.
- Prophylaxis must be started 30–90 min before the surgical procedure. The postoperative regimen has no clear guidelines, but must continue for at least 24 h after the procedure. After this, the wound must be monitored for infection, and therapeutic antibiotics may be instituted only if required. This section is covered in more detail in Chap. 10.1007/978-981-15-1346-6_10, of this book

#### Medication to Prevent Postoperative Gastritis and Vomiting

Preoperative and intraoperative fasting, as well as drugs used in the postoperative period, can induce gastric irritation in the surgical patient. To counter this, drugs that reduce the acidity of gastric secretions may be used. Ranitidine, a H2 blocker, may be used in a dose of 50 mg bd. An alternative drug is Pantoprazole, which may be used in a dose of 40 mg once a day.

Prevention of postoperative nausea and vomiting is an important factor in postoperative care. One of the important drugs used for the management of PONV is Ondansetron, which is given in a dose of 4 mg [23]. This is usually a rescue medication and is not given on a routine basis.

#### Drugs for Thromboprophylaxis

Patients undergoing prolonged surgery or hospitalization may be at increased risk of developing thromboembolic events, namely deep vein thrombosis and pulmonary embolism. Additional risk factors include smoking, pregnancy, oral contraceptives, and malignancy. These patients must be placed on thromboprophylactic drugs. In indicated cases, TED (thromboembolic deterrent) stockings have to be used in the postoperative patients. The guidelines are summarized in Table 12.11 [24].

**Table 12.11:** Thromboprophylaxis guidelines

Risk level	Criteria	Thromboprophylaxis required
Low	Minor surgery, age < 40 yrs., no additional risk factors	Early and persistent mobilization
Moderate	Minor surgery in age 40–60 yrs., with risk factors Major surgery <40 yrs., no risk factors Patients with medical illnesses or burns, with one risk factor Neurosurgical patient with one risk factor	Low molecular weight heparin, <3400 units per day OR intermittent pneumatic compression devices
High	Minor surgery >60 years Major surgery >40 years with risk factors Patients with malignancy Patients with medical illness or burns with two or more risk factors Neurosurgical patient with two or more risk factors Trauma patients with one or more risk factors	Low molecular weight heparin, >3400 units per day OR intermittent pneumatic compression devices
Highest	Patients with multiple risk factors, pelvic and lower extremity trauma or surgery, head injuries	Low molecular weight heparin, >3400 units per day AND intermittent pneumatic compression devices

#### Other Drugs That May be Required Based on the Patients’ Medical History

If the patient has other medical comorbidities, medication that was being taken prior to the procedure may need to be continued or modified. It is best to confer with the patients’ physician to determine the dosage and kind of drugs needed. These have been summarized in Table 12.12.

**Table 12.12:** Additional drugs that may be required in the postoperative period

Medical comorbidity	Drugs to be administered
Diabetes mellitus	Insulin based on insulin sliding scale
Patient with chronic hypertension	Patient’s regular antihypertensive regimen to be restarted within 24 h
Patient on anticoagulants	Low molecular weight heparin for 24 h, after which warfarin should be resumed
Patient on long-term steroids	Hydrocortisone 50 mg three times a day, (equivalent to dexamethasone 2 mg) in addition to patient’s normal dose, for up to 72 h

### Nutritional Status in the Postoperative Period

Maxillofacial surgical procedures provide a unique challenge to the nutritional status in the postoperative period. Pain and edema in the oral region often prevent the patient from taking food comfortably, and there is a tendency to eat less or not at all. In patients with intermaxillary fixation, there is an inability to open the mouth and chew food. In certain kinds of surgery, such as reconstructive flaps involving the oral region, the patient is asked to avoid taking food by mouth at all to prevent the possibility of infection and flap failure in the postoperative period.

It is important, however, that the nutritional status is maintained. Inadequate nutrition has been shown to increase morbidity and mortality and can delay wound healing. It also increases the patient’s susceptibility to infection. In the young, healthy adult patient, nutritional support may not be required, as the body compensates for decreased intake by increased glycogenolysis, gluconeogenesis, lipolysis, and amino acid oxidation. However, in young children, patients with preexisting malnutrition, and patients with wasting diseases, supplementation may be required for even routine procedures. Patients who do not have adequate oral intake for 7–14 days (3–10 days in children) will require support to avoid malnutrition [25].

Nutritional status must be evaluated in the postoperative patient. This usually calls for consultation by a dietician. For long-term patients, nutritional status can also be measured using certain tools. The accepted tool for assessment is the subjective global assessment scale [26].

In patients on intermaxillary fixation, the classic use of the nasogastric tube must be discouraged. Patients may be educated on taking food through the retromolar region, using a feeding tube. NG tubes may be reserved for cases in whom oral feeds are contraindicated to avoid infection. Nutritionally complete formulas (e.g. Ensure) are available for enteral feeds. The patient may be started on 50 ml formula every 4 h, and this may be gradually increased in 50 ml increments until the desired target is achieved. After each feed, the tube must be flushed with 30 ml water to prevent blockage.

In cases of extensive neck surgery, where swallowing may be impaired, percutaneous gastrostomy (PEG) or jejunostomy tubes may be placed. For these tubes, infusion feeds (at the rate of 20 ml/h, increased in 20 ml increments every 4 h) may be given.

Total parenteral nutrition is usually not preferred because it has been linked to higher rates of infectious complications as compared to enteral nutrition. Patients who have complete block of the gastrointestinal system or those who cannot tolerate or retain enteral feeds are candidates for TPN. Dextrose solutions are preferred, with a dose of 10–20 g/kg/day of glucose. This is used in conjunction with amino acid solutions (0.5–3.5 g/kg/day) and lipid emulsions (50 ml/hr) [25].

### Postoperative Mobilization of the Maxillofacial Surgery Patient

After the surgical procedure, early mobilization is recommended for all patients. Early mobilization is believed to enhance recovery by reducing the incidence of postoperative complications. It reduces secretions in the lungs, accelerates peristalsis, and improves venous blood flow to the extremities, thereby preventing thrombophlebitis and deep vein thrombosis [27]. Immobilization increases the risk of complications such as DVT and pressure sores. It can also lead to urinary retention.

For most maxillofacial procedures, the patient may be allowed to sit up with legs dangling 6 h after surgery. The patient may be mobilized within 24 h, and it is recommended that they ambulate every 4–6 h (during waking hours) till discharge. Caution must be employed in patients who have had grafts or flaps taken from the fibula. While the early mobilization protocol must be followed, protected weight bearing may be employed.

For patients who require prolonged bed rest, the use of alternating pressure mattresses or gel mattress overlays must be considered to prevent pressure sores.

Chest physiotherapy forms an important component of postoperative care. The in-hospital patient is prone to increased lung secretions and infections, which may be cleared using chest physiotherapy.

### Management of Complications in the Postoperative Period

#### Sudden Airway Obstruction

Maxillofacial surgery and surgery to the neck carry a risk of edema and hematoma developing in the postoperative period that can compress on the airway. The airway must be monitored closely, both in the immediate postoperative period and during the stay in the ward.

If the patient presents with hypoxia and airway obstruction, the head tilt-chin lift-jaw thrust maneuver must be employed. The airway must be checked manually and cleared of obstruction such as vomitus or blood. If the airway obstruction is at or above the oropharynx, insert an airway (such as Guedel’s) to keep the passage patent. If there is a hematoma compressing the airway, surgical sutures must be removed to allow a release of pressure. In extreme cases, emergency airway procedures such as cricothyroidotomy may need to be performed.

#### Fever in the Postoperative Period

Fever is defined as a rise in body temperature above 38 °C (100.4 °F). Postoperative fever represents a diagnostic challenge for most surgeons. Although most cases of fever are self-limiting, some can be serious and need urgent intervention. The timing of postoperative fever often gives a clue as to its diagnosis and management [28, 29].

##### Immediate Fever (During Surgery or Within the First 24 h).

Fever in the immediate postoperative period is most likely to be an inflammatory response to surgery. The surgical procedure causes *release of pyrogenic cytokines*, which stimulate the anterior hypothalamus to release prostaglandins, causing a rise in body temperature. The extent of fever depends on the amount of tissue trauma, but usually resolves in 24 h. Laboratory and diagnostic workup is not warranted for this kind of fever.

Occasionally, immediate fever can occur due to more serious reasons, and it is important to identify these. *Malignant hyperthermia* is a rare, life-threatening disorder that can manifest in susceptible individuals when they are exposed to inhalational anesthetics, or succinylcholine. There is an immediate rise in body temperature during or up to 1 h after surgery. It may be recognized by an immediate rise in ETCO_2_, tachypnea, tachycardia, and muscle rigidity. Prompt intervention is required to avoid muscle lysis and organ system failure. Treatment involves immediate intravenous Dantrolene sodium (2.5 mg/kg), repeated every 5 min till reversal occurs, or till the maximum dose is reached (10 mg/kg).

If the fever occurs during or immediately *after a blood transfusion*, it is a sign of transfusion reaction. Transfusion of incompatible (mismatched) blood can cause a severe hemolytic reaction, which, in addition to fever, can present with dyspnea, fever, and myoglobinuria. In such cases, the transfusion must be discontinued immediately. Sometimes, febrile reactions can also occur with compatible blood, due to reaction of recipient antibodies with antigens in the transfused blood. This fever will be accompanied by headache, nausea, and vomiting. Slowing the transfusion may suffice, but it must be stopped if the reactions become severe.

*Adverse drug reactions* can rarely cause fever. This is usually a diagnosis of exclusion, and if suspected, all drugs must be discontinued one at a time to identify the offender. If replacement is necessary, a chemically unrelated drug must be used.

##### Early Postoperative Fever (24–48 h After Surgery)

A serious cause of postoperative fever in this time period is *deep vein thromboembolism*. This must be suspected if the patient has known risk factors, such as a history of smoking, malignant disease, prolonged surgery, advanced age, or prolonged immobility after surgery. Diagnosis is made by ultrasound or impedence plethysmography. If present, prompt systemic anticoagulation must be started to avoid fatal pulmonary embolism. The use of Homan’s sign (Pain in the calf on forced dorsiflexion of the foot) is no longer recommended because of the risk of dislodging the thrombus into circulation. A suspected PE may be confirmed with a ventilation-perfusion (V/Q) scan of the lungs.

*Thrombophlebitis* can also cause a rise in body temperature. Any iv line in place for more than 24 h can cause phlebitis. This presents with pain, erythema, and edema at the affected site. The iv line must be removed and replaced, and anti-inflammatory drugs may be given. A topical ointment containing heparin and benzyl nicotinate (thrombophob) may be applied locally.

*Atelectasis* was once thought to be a cause of fever, but it is now believed that fever and atelectasis are unrelated, though they can coexist*. Aspiration pneumonia* is more likely to be a respiratory cause of fever, but it presents 3–5 days after surgery.

##### Delayed Postoperative Fever (After 48 h)

*Surgical site wound infections* can result in fever 3–5 days after surgery. The surgical site must be examined for pain, swelling, and pus discharge if fever occurs during this period. If an infection is present, it must be managed as detailed in the following sections.

*Aspiration pneumonia* can occur if the gastric fluid is aspirated into the lungs, owing to a depressed cough reflex after surgery. The risk increases in patients on maxillomandibular fixation.

##### Fever Beyond Fifth Postoperative Day

Fever beyond the fifth postoperative day is usually a sign of systemic infection and needs a diagnostic workup. The most common infections that can occur are *urinary tract infection* and *upper respiratory tract infection*.

Indwelling urinary catheters are the main source of UTIs. Women are at greater risk because they have a shorter urethra; however, both genders can develop UTI if the catheter is in place for more than 72 h. Concomitant signs such as burning sensation on passing urine may be present. Urine will appear cloudy. Diagnosis is best confirmed by urine culture; empirical antibiotics may be started in the meantime.

Respiratory tract infections can range from sinusitis to hospital-acquired pneumonia. In Hospital Acquired Pneumonia (HAP), chest auscultation may reveal crackles or rales, and diagnosis is made by chest x-rays. Treatment is by empirical antibiotics.

A rare infection that can occur beyond the fifth day is *necrotizing soft tissue infection*. Although this is more common after colorectal surgery, cervical necrotizing infection has been reported after maxillofacial surgery as well [30]. Diagnosis may be made by detecting subcutaneous ‘gas’ on x-rays or CT imaging. Treatment involves the use of broad spectrum antibiotics and fluid resuscitation.

With all systemic infections, blood culture must be done to rule out sepsis. It is also important to monitor the patient’s vitals closely to ensure that the patient does not go into septic shock.

It may be of interest to know that in the literature there is a mnemonic of 6 W’s, with regard to causes of postoperative fever. The W’s being *Waves* (ECG changes, MI), *Wind* (atelectasis, pneumonia), *Water* (UTI), *Wound* (surgical site infection), *Walking* (Venous thromboembolism), *Wonder* drugs(drug-related fever).

**Fig. 12.2:**
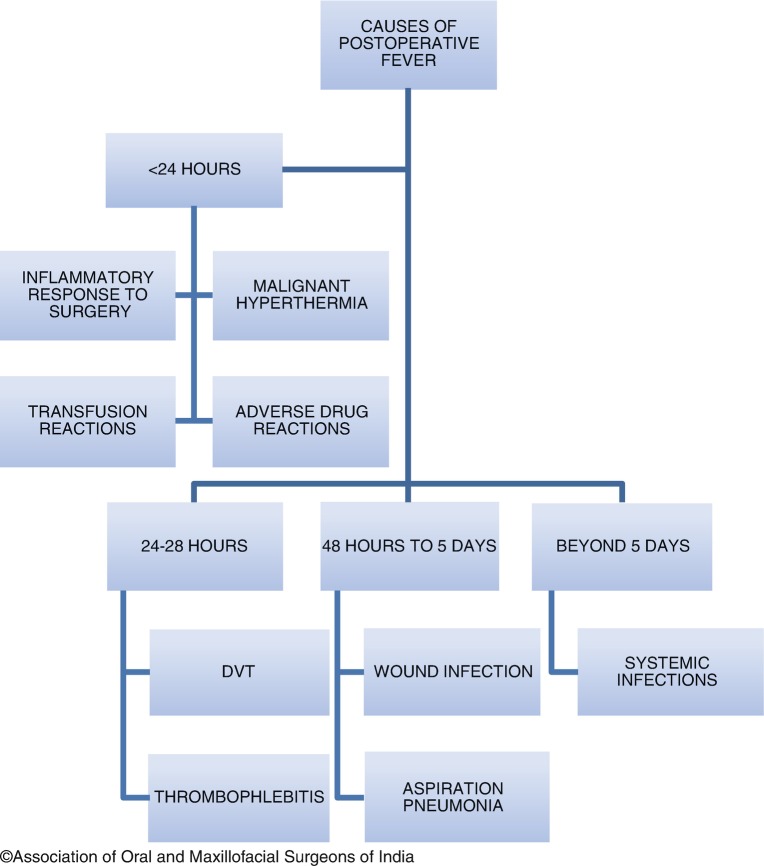
Postoperative fever workup

The workup for postoperative fever is shown in Fig. 12.2.

#### Changes in Pulse and Blood Pressure

Any gross deviation from the normal vital signs must be looked into. Changes in pulse (tachycardia or bradycardia) are usually associated with changes in the other vital signs as well, and these must therefore be evaluated first.

**Fig. 12.3:**
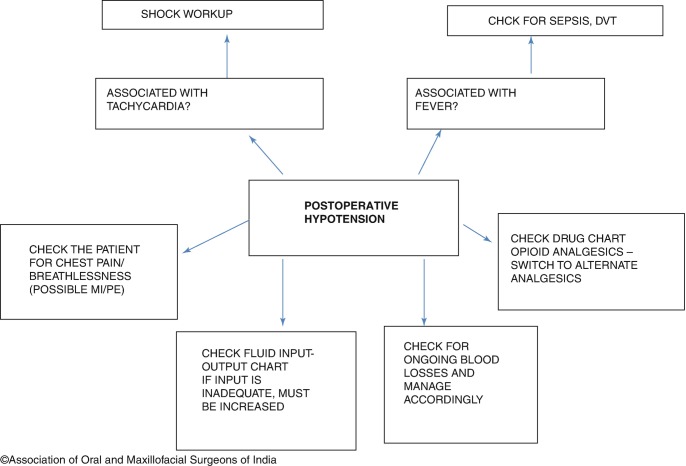
Postoperative hypotension workup

Hypotension in the postoperative period can occur due to several causes [6] (Fig. 12.3). Hypotension is usually the result of reduced plasma volume; this may be due to inadequate fluid resuscitation, or ongoing blood loss. Excessive usage of opioid analgesics may also cause a fall in blood pressure. A myocardial infarction and blood sepsis may also present with hypotension. Hypotension with tachycardia can be a sign of developing shock; which must be treated immediately with a fluid challenge (rapid bolus of fluid). Regardless of the cause, hypotension must be managed by increasing fluid input and supplemented by high-flow oxygen to improve the perfusion.

Raised blood pressure usually occurs in patients who have had preexisting hypertension. If the patient has a history of ischemic heart disease or cerebrovascular disease, it must be managed with appropriate medication, to reduce the risk of developing myocardial infarction or stroke postoperatively. Sometimes, hypertension may occur in healthy adults with no history. In these cases, it could reflect pain and anxiety, or distension of the bowel and/or bladder. Excessive fluid resuscitation may also result in hypertension [6].

#### Changes in Respiration

**Fig. 12.4:**
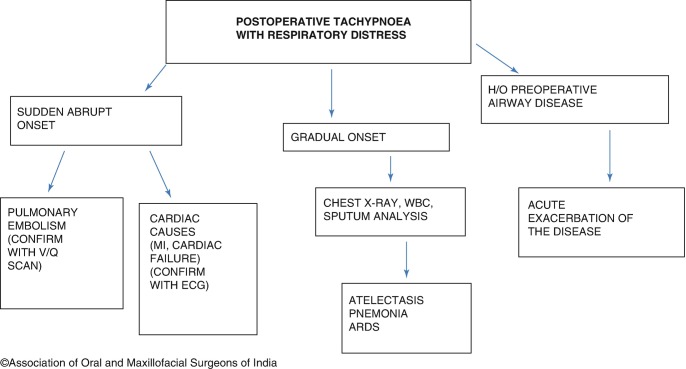
Postoperative respiratory distress workup

Increase in respiratory rate (tachypnea) is usually a sign of respiratory distress, and may be accompanied by decreased oxygen saturation, and use of accessory muscles of respiration [6]. Sudden acute shortness of breath may be a sign of pulmonary embolism. Gradual onset that occurs within 2–5 days of surgery is commonly due to atelectasis. Atelectasis is the collapse of a small segment of the lung and commonly occurs after general anesthesia. Atelectasis after maxillofacial surgery may be obstructive in nature, occurring secondary to epistaxis or mucus secretion. Another reason for the slow onset of respiratory distress is Acute Respiratory Distress Syndrome (ARDS), which can occur secondary to hypovolemia, sepsis, or trauma. Respiratory infection and cardiac causes such as myocardial infarction or cardiac failure can also alter respiratory rate. The various causes are illustrated in Fig. 12.4.

If a person experiences shortness of breath, high-flow oxygen must be started, and a chest x-ray and EKG must be taken. Laboratory tests including cardiac enzymes and arterial blood gases must be performed to ascertain the cause. If sputum is present, it must be sent for culture and gram staining.

#### Postoperative Nausea and Vomiting

This is a common complication that occurs due to activation of the nucleus tractus solitarius and vomiting center by inhalational anesthetics. Patients who undergo maxillofacial surgery, particularly orthognathic surgical procedures, are at increased risk of developing postoperative nausea and vomiting, due to accumulation of blood in the throat. It can affect 20–80% of all patients, the risk being higher in young patients, females in the first 8 days of their menstrual cycle, and obese patients [23]. This complication is best managed symptomatically using antiemetics such as ondansetron and metoclopramide.

#### Oliguria and Acute Renal Failure

The normal urine output is 1 ml/kg/h in infants, 0.5 ml/kg/h in children, and at least 400 ml/day in adults. Any drop below this level is referred to as oliguria and this must be addressed to prevent acute kidney injury (AKI). AKI is diagnosed as oliguria, along with serum creatinine levels 1.5 times above baseline [6]. The various causes of postoperative oliguria and management are outlined in Table 12.13.

**Table 12.13:** Types of postoperative oliguria and management

Type of oliguria/AKI	Cause	Management
Prerenal	Hypovolemia Hypotension	Fluid challenge of 250 ml iv fluid over 1 h Address the cause of hypotension
Renal	Nephrotoxic drugs: NSAIDS, aminoglycosides, steroids Sepsis	Discontinue the offending drug Aggressive treatment with broad spectrum antibiotics and fluid resuscitation
Post-renal	Blocked Foley’s catheter Blocked ureter	Relieve obstruction

## Care of the Surgical Wound Site

### Immediate Care in the Operating Room

Care of the surgical wound site begins even before closure of the wound has been completed. It may be necessary to place drains or catheters within the wound.

#### Surgical Drains

A drain is a device that is intended to evacuate fluids and air from the surgical wound site. By evacuating accumulated pus, blood, and serous fluid, they help prevent infection of the surgical site. By evacuating air, they help eliminate dead spaces, which results in faster wound healing.

The various kinds of drains are summarized in Table 12.14. Drains may be classified as:

- Active drains: These work under negative pressure and actively remove fluids and air from the surgical site.
- Passive drains: They drain fluids passively, and are dependent on gravity. Passive drains may be open or closed.

**Table 12.14:** Types of drains

Type of drain		Example	Indications for use	Image
Active		Jackson-Pratt drain	Inserted into the surgical site to drain blood and serous fluid; to eliminate dead spaces	
		Hemovac		
Passive	Open	Corrugated rubber drain	To drain pus from infected abscesses and anatomical spaces	
		Penrose drain		
	Closed	Nasogastric tube, Foley’s catheter	To drain body secretions (gastric fluid, urine)	

Technique of inserting a surgical drain:

- The drain must be inserted before the last layer of sutures is placed.
- The drain tube has a needle at one end and perforations at the other end.
- The needle is inserted from the deeper edge of the surgical wound, and pulled out through the skin surface, about 2 cm away from the wound margin. The end with the perforations must remain within the surgical site.
- The exit of the needle must be in a dependent area, lower than the incision line.
- The needle is cut off from the tube and discarded safely.
- The drain tube is secured with sutures to the skin surface.
- The bulb or container is deflated completely, to ensure negative pressure, and is then attached to the exit end of the tube.
- Dressing with betadine gauze is done around and over the drain tube.

#### Surgical Site Catheters

Catheters may be inserted directly into the surgical site beneath the skin sutures. These may serve the following purposes:

- Analgesia: An intravenous cannula inserted into the surgical site may be used for delivering local analgesia. Long-acting anesthetics, such as ropivacaine and bupivacaine, are delivered through this into the surgical site. This is especially useful in the iliac crest after harvest of autogenous bone.
- Local antibiotic therapy: Infected spaces or osteomyelitic bone may benefit from local antibiotics that are delivered via catheters.
- Marsupialization: Decompression of a cystic lesion may be followed by catheter insertion into the cystic cavity. This allows continued drainage of fluid from the cyst.

#### Wound Dressing

Once complete suturing of the wound has been completed, appropriate dressing of the wound site must be carried out. Various types of dressing material are available [31]. These are detailed in the Chap. 10.1007/978-981-15-1346-6_11 on soft tissue injuries and management.

### General Guidelines for Postoperative Wound Care [32]

- Aim to leave the wound undisturbed for at least 48 h after surgery.
- Premature removal of the wound dressing may, however, be required in certain situations. These include:
- Excess exudate or blood soaking through the dressing.
- Suspicion that the wound site is infected (e.g. Postoperative fever with no other attributable cause).
- The dressing is no longer serving its purpose (e.g. Falling off).
- If the wound dressing is being changed, check if there is excess exudate or devitalized tissue that may delay wound healing. If these are present, the wound must be cleansed. This is done by gentle irrigation of saline (for the first 48 h) or clean tap water (after 48 h) using a syringe. The wound must never be swabbed with gauze, as this can delay healing.

#### Postoperative Care of Drains and Catheters

- The drain must be monitored every 4 h and more frequently if the discharge from the wound is excessive.
- The drain container must be evacuated at least once every day.
- Once the drain fluid collection goes below 25–50 ml/day, the drain may be removed.
- Surgical site catheters must be removed by the third postoperative day.

### Surgical Site Complications

#### Postoperative Bleeding, Hematoma, and Seroma

Bleeding after surgery is classified as primary (occurs during or immediately after the surgical procedure), reactionary (occurs after few hours, possibly due to slipped ligatures), and secondary (occurs after few days, commonly due to infection). Active, ongoing bleeding is referred to as hemorrhage.

Hematoma refers to a clotted collection of blood below the tissues, which occurs due to damage to vessel walls. Most hematomas are self-limiting. Larger hematomas can be treated with ice packs or compression dressings. Analgesics may be given if the swelling is painful. Hematomas in the submandibular and neck region, or other regions which can potentially compress the airway, must be drained surgically.

Seromas are collections of serous fluid that generally develop 5–7 days after surgery. They are more common in extensive surgeries, particularly in neck dissection, where lymph nodes have been removed. Small seromas resolve over time. If the seroma is large and painful, the fluid may be aspirated and a pressure dressing can be applied.

#### Infected Wound

As maxillofacial procedures are clean-contaminated surgeries, they carry a higher risk of developing infection as compared to clean surgeries. Signs and symptoms of an infected wound include:

- Unexplained fever 3–5 days after surgery.
- Localized pain at the wound site.
- Erythema around the wound.
- Wound dehiscence, with pus discharge from the wound.

If the wound appears infected, a few sutures must be removed and pus must be drained out. A swab must be sent for culture and sensitivity testing, and the patient must be started on empirical antibiotics. The patient must be monitored closely for signs of systemic infection. The infected wound can be treated by local debridement and antibiotic irrigation.

#### Wound Dehiscence

This generally occurs as a result of one of the above complications, most commonly subacute infection. Sometimes, however, it may simply be the result of excessive wound tension due to inadequate tissue undermining. If the wound is uninfected less than 24 h postoperatively, re-suturing may be attempted. Otherwise, the wound is best left to heal by secondary intention. Resistant wound dehiscence may lead to orocutaneous communications and may become difficult to handle. The decision to either allow these to granulate or surgically provide a cover depends on individual scenarios.

## Postoperative Care for Specific Types of Surgeries

While the general rules apply to all kinds of surgeries, there may be extra measures which are required in each specific surgery type. These are outlined below [2].

### Postoperative Care for the Trauma Patient

- In the immediate postoperative period, the airway must be monitored. This may get compromised due to bleeding or edema.
- For fractures of the zygoma and orbit, monitoring for retrobulbar hemorrhage is important. This can be diagnosed by the three Ps—pain (in the orbit), proptosis, and pupillary defects [33]. The postoperative care team is generally required to check papillary reflexes on an hourly basis for the first 6 h which can be relaxed to once in 2 h for the next 6 h. Any impending sign of diminishing vision or pupillary inactivity is brought to the notice of the consultant and steps initiated accordingly.
- Assess and document paresthesia along any nerves involved in the fractured segment. This includes the inferior alveolar nerve for mandibular fractures and infraorbital nerve for zygomaticomaxillary complex fractures.
- The accuracy of reduced fractures must be checked on the first postoperative day using radiographs. For fractures involving the dentoalveolar segments, occlusion may be used as a guide.

### Postoperative Care for Orthognathic Surgery Patients

- As with trauma patients, airway compromise due to bleeding and edema must be monitored.
- Considerable facial swelling is common. Ice packs and systemic steroids can help reduce swelling.
- Occlusion must be checked on the first postoperative day, and elastics must be placed if required. Class II elastics are placed from the upper anterior teeth to lower posterior teeth. Class III elastics are placed from lower anteriors to upper posterior teeth.

### Postoperative Care for Oncology and Reconstruction Patients

- These patients usually undergo extensive surgery that lasts for hours, and will therefore need intensive monitoring in the postoperative period. Tracheostomy care may be required (See Sect. 12.1.1).
- Most of these patients will be unable to feed properly till wound healing is complete. Ryle’s tube or PEG insertion must be done on the day of surgery or the first postoperative day.
- Patients must be on DVT prophylaxis. The malignancy, prolonged surgery, and in-hospital stay increase the risk of the patient developing DVT.

### Flap Monitoring

- Reconstructed flaps must be monitored for vitality. If the blood supply to the flap is lost due to thrombi, it may be salvageable within the first 48 h. Periodic monitoring ensures prompt surgical intervention if the need arises.
- Flaps may be monitored using a handheld Doppler. Capillary refill may be assessed for flaps which are not buried. The needle prick test is simple, but must be done only to confirm congestion or loss of vitality.
- Flaps must be monitored hourly for the first 24 h, and 4 hourly for the next 48 h.

## Postoperative Complications Specific to each Type of Surgery

Table 12.15 lists the complications that may be encountered with each specific surgery.

**Table 12.15:** Specific postoperative complications

Type of surgery	Complication	Management
Trauma	Malunion/nonunion Ptosis, diplopia, enophthalmos (orbital and ZMC repair) Persistent paresthesia	Reopen, remove cause of malunion, induce bleeding, use of graft Secondary surgical correction Palliative
TMJ surgery/condylar fractures	Frey’s syndrome (gustatory sweating) Sialocele, salivary fistula Facial nerve palsy	Botox injection, surgical intervention Debride the fistula lining and keep the area clean till healing occurs. Temporary—Palliative care Permanent—Facial nerve reanimation
Orthognathic surgery	Condylar malpositioning leading to anterior open bite (BSSO) Inferior alveolar nerve paresthesia Alar base widening (maxillary surgery) Nasal septal deviation Aseptic necrosis of segment Malocclusion	Re-surgery, removal of plates, repositioning the condyle and re-fixation Palliative management Alar cinching Septoplasty Removal and reconstruction Orthodontic treatment
Neck dissections	Chyle leak Shoulder syndrome (spinal accessory nerve damage) Paresthesia of sensory nerves, e.g. greater auricular, lingual nerves Carotid blowout	Chyle duct repair Physiotherapy Palliative Surgical emergency, immediate repair needed.

## Criteria for Discharging the Patient

Prolonged hospital stay increases the patient’s risk of developing nosocomial infections. Therefore, the patients must be discharged as soon as feasible. Criteria for discharge are as follows:

- Patient is hemodynamically stable, and all vitals are stable.
- Patient can take nutrition independently or with the aid of a caregiver.
- Wounds are healing well with no signs of infection.

Discharge summary: This is a record given to the patient, detailing the diagnosis, procedure performed, and any implants that were used. Instructions and medications to be taken must also be noted.

Follow-up appointments must be made for review. In case facial and neck incisions were placed, an appointment for suture removal must be given.

## Conclusion

Postoperative care requires a thorough understanding of the patient, the procedure they have undergone, and the expected outcome. Surgeons must realize that more often it may be the first experience for a patient in going under the knife and lying at the ICU or ward. Hence, a perfect coherence of scientific and psychological views is required of the surgical team at all levels. The chapter emphasizes the need for comprehensive management of the maxillofacial patient at the ward after their procedure. The reader is expected to understand that outcome depends, but not ends at operating room or ward/ICU.
